# Differential mitochondrial proteomic analysis of A549 cells infected with avian influenza virus subtypes H5 and H9

**DOI:** 10.1186/s12985-021-01512-4

**Published:** 2021-02-18

**Authors:** Yuting Yang, Yun Zhang, Changcheng Yang, Fang Fang, Ying Wang, Haiyan Chang, Ze Chen, Ping Chen

**Affiliations:** 1 College of Life Sciences, Hunan Normal University, Changsha, 410081 Hunan China; 2 Shanghai Institute of Biological Products, Shanghai, 200052 China

**Keywords:** Influenza virus, Mitochondria, Two-dimensional electrophoresis, Proteomics

## Abstract

**Background:**

Both the highly pathogenic avian influenza (HPAI) H5N1 and low pathogenic avian influenza (LPAI) H9N2 viruses have been reported to cross species barriers to infect humans. H5N1 viruses can cause severe damage and are associated with a high mortality rate, but H9N2 viruses do not cause such outcomes. Our purpose was to use proteomics technology to study the differential expression of mitochondrial-related proteins related to H5N1 and H9N2 virus infections.

**Methods:**

According to the determined viral infection titer, A549 cells were infected with 1 multiplicity of infection virus, and the mitochondria were extracted after 24 h of incubation. The protein from lysed mitochondria was analyzed by the BCA method to determine the protein concentration, as well as SDS-PAGE (preliminary analysis), two-dimensional gel electrophoresis, and mass spectrometry. Differential protein spots were selected, and Western blotting was performed to verify the proteomics results. The identified proteins were subjected to GO analysis for subcellular localization, KEGG analysis for functional classification and signaling pathways assessment, and STRING analysis for functional protein association network construction.

**Results:**

In the 2-D gel electrophoresis analysis, 227 protein spots were detected in the H5N1-infected group, and 169 protein spots were detected in the H9N2-infected group. Protein spots were further subjected to mass spectrometry identification and removal of redundancy, and 32 differentially expressed proteins were identified. Compared with the H9N2 group, the H5N1-infected group had 16 upregulated mitochondrial proteins and 16 downregulated proteins. The differential expression of 70-kDa heat shock protein analogs, short-chain enoyl-CoA hydratase, malate dehydrogenase, and ATP synthase was verified by Western blot, and the results were consistent with the proteomics findings. Functional analysis indicated that these differentially expressed proteins were primarily involved in apoptosis and metabolism.

**Conclusions:**

Compared with their expression in the H9N2 group, the differential expression of eight mitochondrial proteins in the H5N1 group led to host T cell activation, antigen presentation, stress response, ATP synthesis and cell apoptosis reduction, leading to higher pathogenicity of H5N1 than H9N2.

**Supplementary Information:**

The online version contains supplementary material available at 10.1186/s12985-021-01512-4.

## Background

H5N1, an influenza A virus (IAV), is a highly pathogenic avian influenza (HPAI)virus [1, 2]. It was first isolated and identified in domestic geese in Guangdong Province, China, in 1996. The spread of H5N1 avian influenza illness in poultry populations increases the risk of human infection [3]. In May 1997, the first human H5N1 virus infection occurred in the Hong Kong Special Administrative Region of China: 18 people were infected and 6 died. As of 2019, H5N1 IAV had migrated to at least 17 countries, caused 861 confirmed infections and 455 deaths in humans [4]. To date, more than 850 cases of human infection with the H5N1 virus have been confirmed, with a mortality rate of approximately 60% [5]. H9N2, another IAV, is a low pathogenic avian influenza (LPAI) virus [6]. Individuals infected with LPAI H9N2 viruses generally have a mild upper respiratory tract illness, with only one death to date.

Influenza virus induces caspase-dependent apoptosis by activating caspase-3 [7]. Apoptosis is divided into the extrinsic and intrinsic pathways. The intrinsic apoptotic pathway engages caspases via members of the BCL-2 protein family and mitochondria in response to severe cellular damage or stress [8]. Mitochondria also play a leading role in the release of many important apoptosis-inducing molecules due to mitochondrial outer membrane permeabilization (MOMP) [9]. According to a whole-cell proteomic study of A549 cells infected with avian influenza virus H7N9 and influenza virus H1N1, some differentially expressed proteins are localized to mitochondria [10]. Influenza virus is a high-risk virus that poses a great threat to human health and the economy. According to their virulence level, IAVs are divided into LPAI and HPAI viruses. Most avian influenza viruses are LPAI viruses, which can produce subclinical infections in poultry or occasionally cause mild respiratory diseases, reduced egg production and low mortality rates [11]. HA subtype H5 and H7 LPAI viruses can mutate into HPAI variants. It is represented by H5N1 and H7N7, leading to severe progressive disease and high bird mortality [12]. HPAI viruses can seriously affect animal health and have an economic impact on the commercial poultry industry. In addition, some strains can be transmitted to humans [13].

We selected LPAI H9N2 virus-infected A549 cells as the control group and HPAI H5N1 virus-infected A549 cells as the test group in this study. Two-dimensional difference gel electrophoresis (2D) and MALDI-TOF tandem mass spectrometry (MS/MS) were applied to investigate the differences in the host proteome after infection with these two influenza virus strains and to explore the different pathogenic mechanisms of A/Chicken/Jiangsu/07/2002 (H9N2) and A/Chicken/Henan/12/2004 (H5N1) in infected human cells.

## Methods and materials

### Cell culture

A549 adenocarcinoma cells were purchased from the Cell Resource Center of the Shanghai Academy of Sciences, Chinese Academy of Sciences. A549 adenocarcinoma cells were cultured in F-12K Nutrient Mixture (GIBCO, Grand Island, NY, USA) at pH 7.2, supplemented with 10% fetal bovine serum (GIBCO) and penicillin (100 U/mL)/streptomycin (100 µg/mL) and grown in a cell culture incubator at 37 °C, under conditions of 5% CO_2_ and saturated humidity.

### Virus infection and tissue culture infective Dose_50_ (TCID_50_)

A/Chicken/Jiangsu/07/2002 (H9N2) and A/Chicken/Henan/12/2004 (H5N1) were obtained from the Wuhan Institute of Virology, Chinese Academy of Sciences. A549 cells (80% confluent) were infected with H5N1 and H9N2 viruses at a multiplicity of infection (MOI) of 1 for 1 h, and viral growth medium (serum-free complete F-12K supplemented with 2 µg/mL of TPCK-treated trypsin) was added at 24 h.

The virus was diluted tenfold with a virus dilution solution (phosphate-buffered saline + 0.1%BSA) [14], and 100 μL of virus solution was added to the cells, followed by a 1-h incubation period in a cell culture incubator at 37 °C, with 5% CO_2_ and saturated humidity. After the virus and cells were incubated for 1 h, the virus–serum mixture was replaced with 5 mL of viral growth medium. After culture for 72 h [15], the median TCID (TCID_50_) was calculated by the method of Reed and Muench [16].

### Extraction of mitochondrial proteins from virus-infected cells

Virus-infected cells were washed once with precooled PBS, and lysis buffer (25 mM mannitol, 0.5 mM EGTA, 5 mM HEPES, 0.1% BSA [w/v]) was added. The cells were scraped and placed in a homogenizer for homogenization. The cell sample was centrifuged at 600 × g for 5 min at 4 °C, and the supernatant was collected. The supernatant obtained by centrifugation in the previous step was centrifuged at 10,300 × g for 10 min at 4 °C, and the precipitate was mitochondrial protein [17]. The previous centrifugation step was repeated, and the precipitate was combined with that obtained in the previous step to further maximally enrich mitochondrial protein. The obtained mitochondrial protein was suspended in acetone precooled to − 20 °C, stored at − 20 °C for 12 h or more, and centrifuged at 8750 × g for 35 min at 4 °C. The supernatant was then removed, and the centrifugation step was repeated once. On an ultraclean work bench, samples were naturally air-dried on ice, and an appropriate amount of lysate was added to fully dissolve the precipitate. Protein was further dissolved by vortexing [18, 19].

The sample was collected and the protein concentration was determined using a bicinchoninic acid (BCA) protein assay kit (Sangon Biotech, Shanghai, China) according to the manufacturer’s instructions. The samples were then aliquoted and stored at − 80 °C until subsequent use.

### Two-dimensional (2-D) gel electrophoresis

Rehydration solution (8 M urea; 2 M thiourea; 4.0% [w/v] CHAPS; 20 mM Tris base; 20 mM DL-dithiothreitol; 0.5% [v/v] pH 3–10 amidine, and 10% [v/v] bromophenol blue) was added to the protein sample. For isolation of proteins by isoelectric focusing (IEF), a salt bridge was formed at the two poles of the electrophoresis tank, the supernatant of the centrifuged protein sample was added uniformly to the electrophoresis tank, and the isoelectric focusing strip (17 cm, pH 3–10) was removed from the − 20 °C freezer and equilibrated to room temperature. The sample was added to the electrophoresis channel, and the appropriate amount of mineral oil was added to cover the strip. The program was set as follows: 50 V for 14 h, passive rehydration; 500 V for 1 h, linear; 1000 V for 1 h, rapid; 5000 V for 1 h, rapid; 8000 V for 1 h, linear; and rapid ramping to 8000 V for 60,000 Vh [17]. After isoelectric focusing, strips were equilibrated in equilibration buffer (6 M urea, 20% glycerol, 2% SDS, 25 mM Tris–HCl [pH 8.8]) containing 0.2% (w/v) dithiothreitol for 15 min and then in the same buffer containing 3.0% (w/v) iodoacetamide and 0.175% (v/v) bromophenol blue for 15 min. Separation in the second direction was performed via 12.5% SDS-PAGE under a constant current of 25 mA, and gels were stained with Coomassie Brilliant Blue G-250. After decolorization, analysis was performed using ImageMaster software to match gel spots, and gray values that were significantly different (gray value ≥ 2.0-fold) between the H9N2-infected and H5N1-infected groups were selected for MS analysis.

### In-gel trypsin digestion, MS and data searching

The sample was mixed in an equal ratio with 10 mg/mL α-cyano-4-hydroxcinnamic acid, directly spotted onto a spotting plate, and allowed to dry at room temperature. Peptide mass spectra were obtained with a 5800 MALDI TOF/TOF mass spectrometer (AB SCIEX, Foster City, USA). The MS/MS data of the peptide mass fingerprint (PMF) were submitted to the online software Mascot (Matrix Science, Boston, MA, USA) for identification according to the NCBIProt database. The parameters were as follows: taxonomy, Homo sapiens; enzyme, trypsin; allowed missed cleavages, two; variable modification, oxidation (M); fixed modification, carbamidomethylation (C); and number of peptide charges, + 1. The peptide and fragment mass tolerances were set at ± 1.2 Da and ± 0.6 Da, respectively. The data format was selected as Mascot generic, and the instrument was selected as MALDI-TOF-TOF. Proteins with a score greater than 30 were regarded as trustworthy proteins[17].

### Western blot analysis

The extracted total cell lysate and mitochondrial proteins of H5N1 and H9N2 virus-infected A549 cells were quantified with a BCA kit (Sangon Biotech, Shanghai, China). Mitochondrial proteins (40 µg) and total cell lysate (40 µg) were separated by 12% SDS-PAGE and transferred to nitrocellulose membranes (BBI Life Sciences). After blocking with 5% (w/v) skim milk in TBST (50 mM Tris [pH 8.0], 150 mM NaCl, 0.1% [v/v] Tween-20) for 1 h at 37 °C, membranes were incubated separately overnight at 4 °C with rabbit monoclonal or polyclonal antibodies against ECHS1 (ab170108), MDH2 (ab181873) (Abcam), ATP5F1 (15999-1-ap), HSPA1L (13970-1-ap), BAX (50599-2-Ig), and Caspase 3 (66470-2-Ig) (Proteintech). After three washes with TBST, membranes were incubated with horseradish peroxidase (HRP)-conjugated goat anti-rabbit IgG or HRP-conjugated goat anti-mouse IgG (used at a 1:5000 dilution, Proteintech) for 1 h at room temperature and were then washed three times with TBST. The immunoreactive protein bands were detected using enhanced chemiluminescence reagent (ECL; Advansta, CA, USA), with TOM40 (18409-1-ap) (Proteintech) and β-actin (66009-1-Ig) (Proteintech) as the loading controls.

### Bioinformatics analysis

The Omicsbean online analysis software (http://www.omicsbean.cn) was used with the accession number of each identified protein to perform Gene Ontology (GO) classification and Kyoto Encyclopedia of Genes and Genomes (KEGG) analysis for signaling pathways. The accession number of each identified protein was submitted for STRING (https://string-db.org) analysis for functional protein association network construction. Regular functional analysis was performed using the tools in the Swiss-Prot database (http://uniprot.org) [17, 20].

### Statistical analysis

The statistical significance of differences between groups was determined by using a paired, nonparametric Student’s T-test. *P* < 0.05 was considered statistically significant. The experiment was repeated three times.

## Results

### 2D Screening and identification of differentially expressed proteins

Mitochondrial protein extracts (1500 µg) from A549 cells infected with H5N1 or H9N2 influenza viruses were loaded onto 2-D gels. Two-dimensional gel electrophoresis showed 227 protein spots in the H5N1 group (Figure S1A) and 169 protein spots in the H9N2 group (Figure S1B). Eight differentially expressed protein spots from the mitochondria are also illustrated in an enlarged format (Figure S1C). After further MS identification and removal of redundancy, 32 differentially expressed proteins were identified. Compared with the H9N2-infected group, the H5N1-infected group had 16 upregulated mitochondrial proteins and 16 downregulated proteins (Tables 1, 2). Upregulated 70-kDa heat shock protein 1-like (HSPA1L) is located in the mitochondrial matrix, and short-chain enoyl-CoA hydratase (ECHS1) is located in the inner membrane of the mitochondria. Among these downregulated proteins, stress-70 protein, malate dehydrogenase (MDH2), mitochondrial membrane ATP synthase (ATP5F1), and stomatin-like 2 protein are located in the mitochondrial inner membrane, while peroxiredoxin 5 and 60-kDa heat shock protein (HSP60) are located in the mitochondrial matrix.

**Table 1:** Summary of downregulated proteins in A549 cells infected with influenza A H5N1 virus compared to H9N2-infected cells at 24 hpi (r > 2, *p* < 0.05)

Spot no.^a^	Accession No.^b^	Gene	Protein name	MW (Da)	pI	Score^c^
1	P04264	KRT1	Keratin, type II cytoskeletal 1	66,170	8.15	120
2	P10809	HSPD1	60-kDa heat shock protein, mitochondrial	61,187	5.7	69
3	B4DEF7	N /A	cDNA FLJ60062, highly similar to 78-kDa glucose-regulated protein	30,458	5.77	68
4	Q59FC6	N /A	Tumor rejection antigen (Gp96) 1 variant	66,140	5.08	46
5	V9HWE1	HEL113	Epididymis luminal protein 113	53,676	5.24	156
6	Q7L4M3	KRT 8	KRT8 protein	30,802	5.05	57
7	P07237	P4HB	Protein disulfide-isomerase	57,146	5.96	60
8^*^	P06576	ATP5F1B	ATP synthase subunit beta, mitochondrial	56,525	5.26	130
9	Q9UJZ1	STOML2	Stomatin-like protein 2, mitochondrial	38,839	4.45	120
10	P35908	KRT2	Keratin, type II cytoskeletal 2 epidermal	65,678	8.07	47
11	P13645	KRT10	Keratin, type I cytoskeletal 10	58,994	5.13	49
12	A8K401	PHB	Prohibitin, isoform CRA_a	29,843	4.14	175
13	P30044	PRDX5	Peroxiredoxin-5, mitochondrial	17,611	9.02	50
14	Q9BYX7	POTEKP	Putative beta-actin-like protein 3	42,331	5.91	53
15^*^	Q6FHZ0	MDH2	Malate dehydrogenase, mitochondrial	35,965	8.92	63
16	P38646	HSPA9	Stress-70 protein, mitochondrial	73,967	6.03	52

^a^Spot No. is the unique sample spot protein number

^b^Accession No is the MASCOT result of MALDI-TOF/TOF searched from the NCBI database

^c^Protein score (based on combined MS and MS/MS spectra) and best ion score (based on MS/MS spectra) were from MALDI-TOF/TOF identification

^*^Indicating that the differential proteins were validated by western blot analysis

**Table 2:** Summary of upregulated proteins in A549 cells infected with influenza A H5N1 virus compared to H9N2-infected cells at 24 hpi (r > 2, *p* < 0.05)

Spot no.^a^	Accession No.^b^	GENE	Protein name	MW (Da)	pI	Score^c^
17^*^	P30084	ECHS1	Enoyl-CoA hydratase, mitochondrial	31,716	6.05	68
18	P02768	ALB	Serum albumin	47,098	5.92	64
19	P35527	KRT9	Keratin, type I cytoskeletal 9	62,255	5.14	72
20	Q9HC85	MB2	Metastasis related protein	10,414	5.16	35
21	P11021	HSPA5	Endoplasmic reticulum chaperone BiP	71,002	5.23	155
22	AIZ70879	N/A	immunoglobulin heavy chain variable region, partial	8328	11.84	42
23^*^	P34931	HSPA1L	heat shock 70-kDa protein 1-like	77,913	7.55	64
24	A0A1L1UHR1	HTL-T-186e	Homo sapiens sperm binding protein 1aing mRNA	30,868	8.62	46
25	P09651	HNRNPA1	Heterogeneous nuclear ribonucleoprotein A1	34,289	9.27	58
26	P22626	HNRNPA2B1	Heterogeneous nuclear ribonucleoproteins A2/B1	36,041	8.67	52
27	P08670	VIM	vimentin isoform 1, partial	53,676	5.06	56
28	Q8N1N4	KRT78	Keratin, type II cytoskeletal 78	57,728	5.79	51
29	Q86Y46	KRT73	Keratin, type II cytoskeletal 73	42,270	8.42	51
30	D9YZU9	HBG2	TPA: globin B2	16,173	6.64	50
31	P68871	HBB	Beta globin	11,534	5.9	46
32	P69905	HBA1	Hemoglobin subunit alpha	15,174	8.73	65

^a^Spot No. is the unique sample spot protein number

^b^Accession No is the MASCOT result of MALDI-TOF/TOF searched from the NCBI database

^c^Protein score (based on combined MS and MS/MS spectra) and best ion score (based on MS/MS spectra) were from MALDI-TOF/TOF identification

^*^Indicating that the differential proteins were validated by western blot analysis

### Gene ontology analysis of differentially expressed proteins

**Fig. 1:**
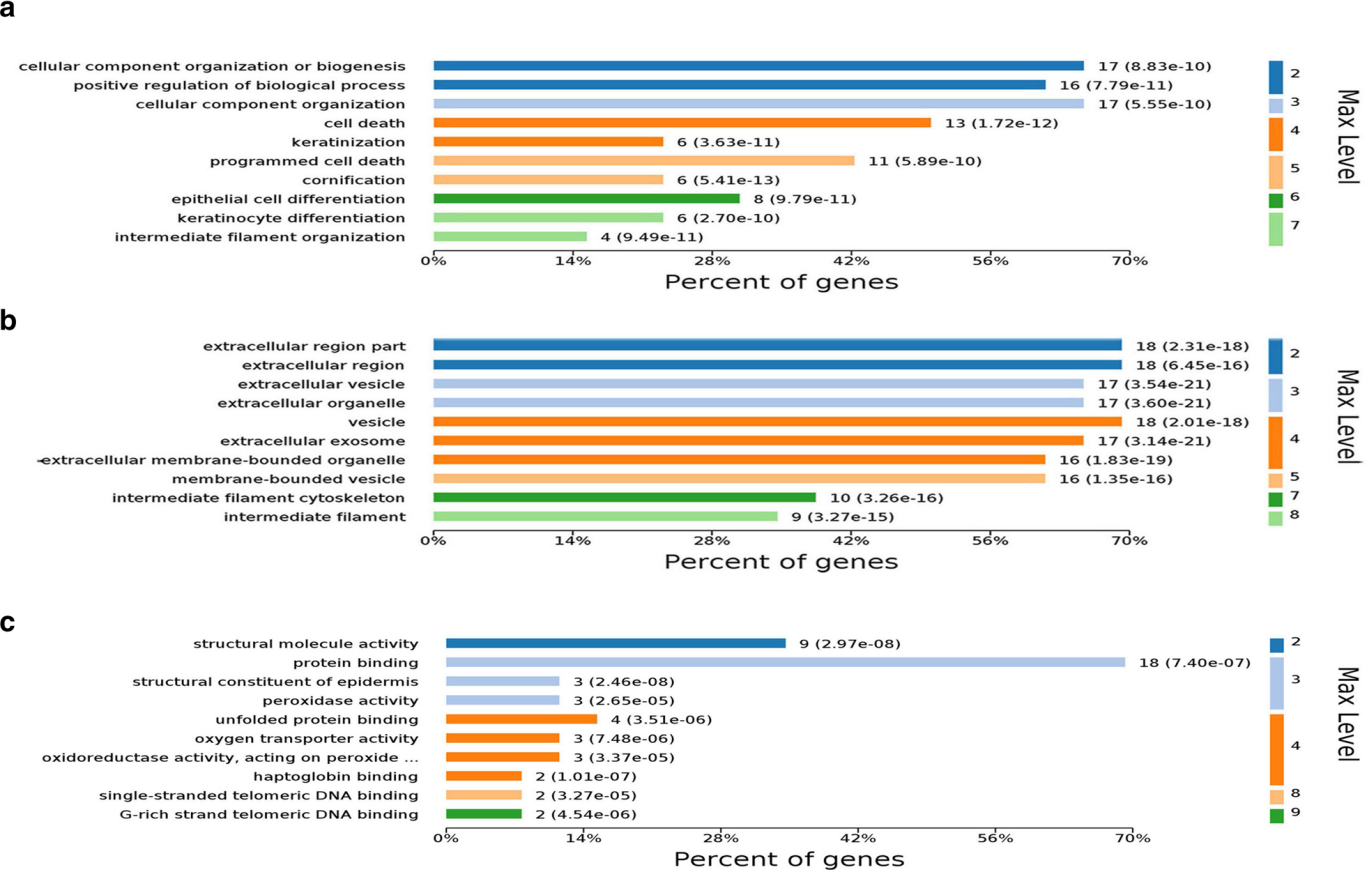
Differentially expressed protein GO annotation. **a** Biological Process. **b** Cellular Component. **c** Molecular Function

The proteins corresponding to the 32 differential protein spots were subjected to GO analysis. Only 26 of the 32 differentially expressed proteins were annotated through data analysis. The main biological processes involving these differentially expressed proteins are shown in Fig. 1a; specifically, 65.38% (17 proteins) were cellular components or involved in biogenesis, 61.53% (16 proteins) were involved in the positive regulation of biological processes, 50% (13 proteins) were associated with cell death, and 42.3% (11 proteins) were involved in programmed cell death. These findings indicate that the differences in the apoptotic process may be the cause of the differences in the host cells infected by different subtypes of influenza viruses. The cellular components are shown in Fig. 1b, with 65.38% (17 proteins) belonging to the organelle component. The molecular functions of the differentially expressed proteins are shown in Fig. 1c; 84.61% (22 proteins) were involved in binding, including protein binding, DNA binding and other molecular binding functions. These findings indicate that the binding proteins have an important role in host infection with different subtypes of influenza viruses.

### KEGG pathway analysis of differentially expressed proteins and construction of the protein interaction network diagram

**Fig. 2:**
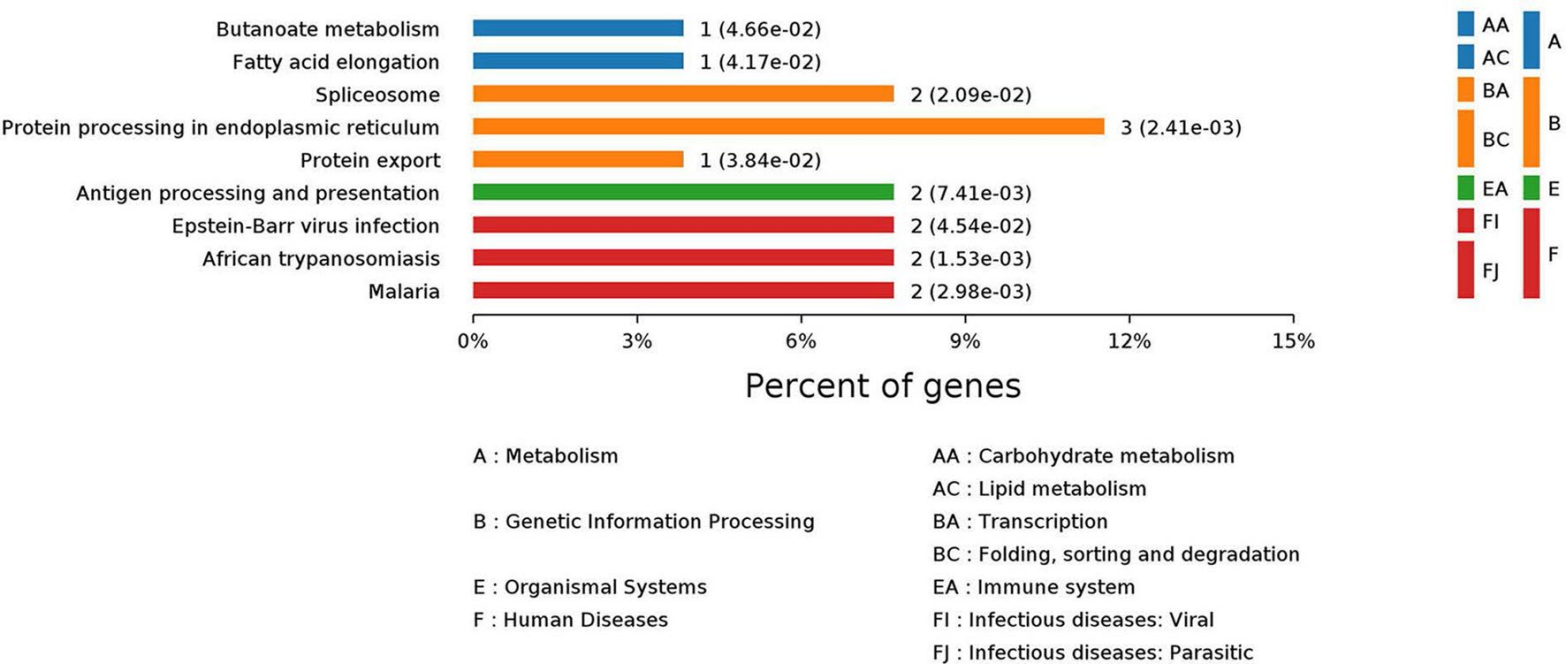
Analysis of differentially expressed proteins in KEGG signaling pathways

KEGG analysis of signaling pathways involving the differentially expressed proteins was conducted. The differentially expressed proteins were involved in 37 signaling pathways, 9 of which were significantly different (*P* < 0.05) (Fig. 2). A total of 7.69% were involved in metabolic processes, 3.85% of which participated in carbohydrate metabolism, 3.85% in lipid metabolism, and 23.08% in genetic information processes. Of the 23.08%, 7.69% participated in the transcription process; 11.54% participated in protein folding, classification, and degradation processes; 7.69% were involved in antigen processing and presentation in the immune system; 7.69% were involved in virus infection; and 7.69% participated in parasitic infection. These findings indicate that the differential proteins involved in antigen processing and presentation of the immune system and viral infectious diseases have important roles in the differences between host cells infected with different subtypes of influenza virus.

**Fig. 3:**
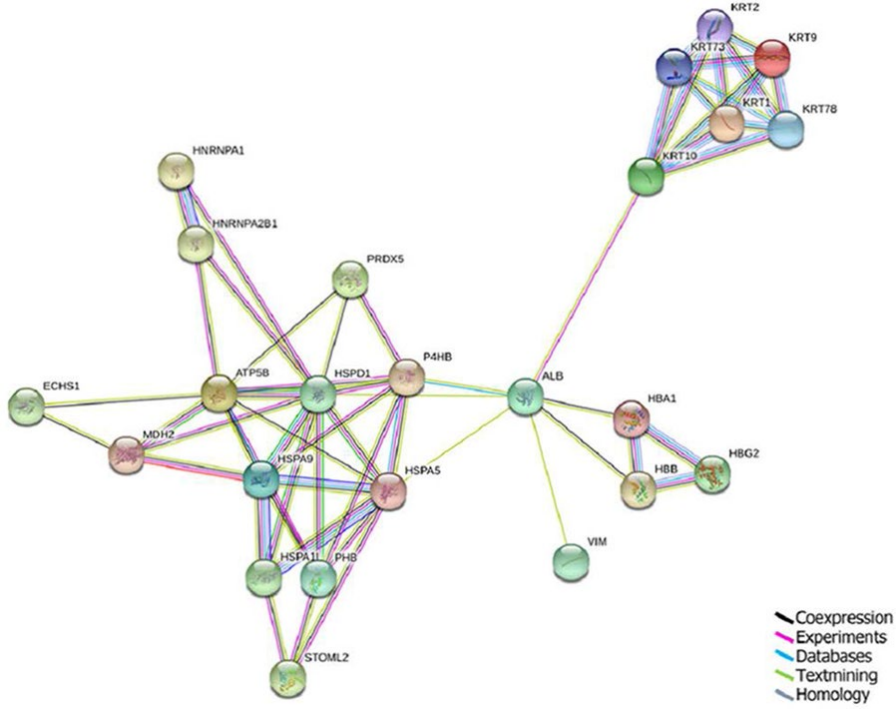
Protein–protein interaction (PPI) network diagrams at 24 h were a constructed by STRING software. Colored lines denote interactions. The black line represents coexpression, the purple line represents experiments, the cyan line represents databases, the green line represents text mining, and the blue line represents homology

Protein–protein interaction analysis was performed on the differentially expressed proteins using STRING online software. From this analysis 24 of the 32 differentially expressed proteins were identified, and the results are shown in Fig. 3. The overall set of differentially expressed proteins is divided into three sections, and the albumin (ALB) protein connects these three sections. The ALB protein is involved in mitochondrial reactive oxygen species (ROS) production [21]. As a node of the regulatory network, it may play an important role in the virus infection process. Moreover, the proteins essentially interact with each other. The proteins ATP5FB, MDH2, ECHS1, and HSPA1L are located in the key site of the network and play crucial roles in the difference in virulence between H5N1 and H9N2.

### Western blot of differentially expressed proteins

**Fig. 4:**
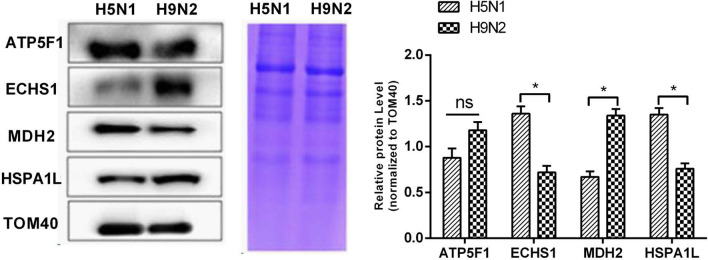
Effects of H9N2 and H5N1 infection on ATP5F1, ECHS1, HSPA1L and MDH2 mitochondrial protein expression in A549 cells.*, *p* < 0.05 versus H9N2-infected group; n = 3

**Fig. 5:**
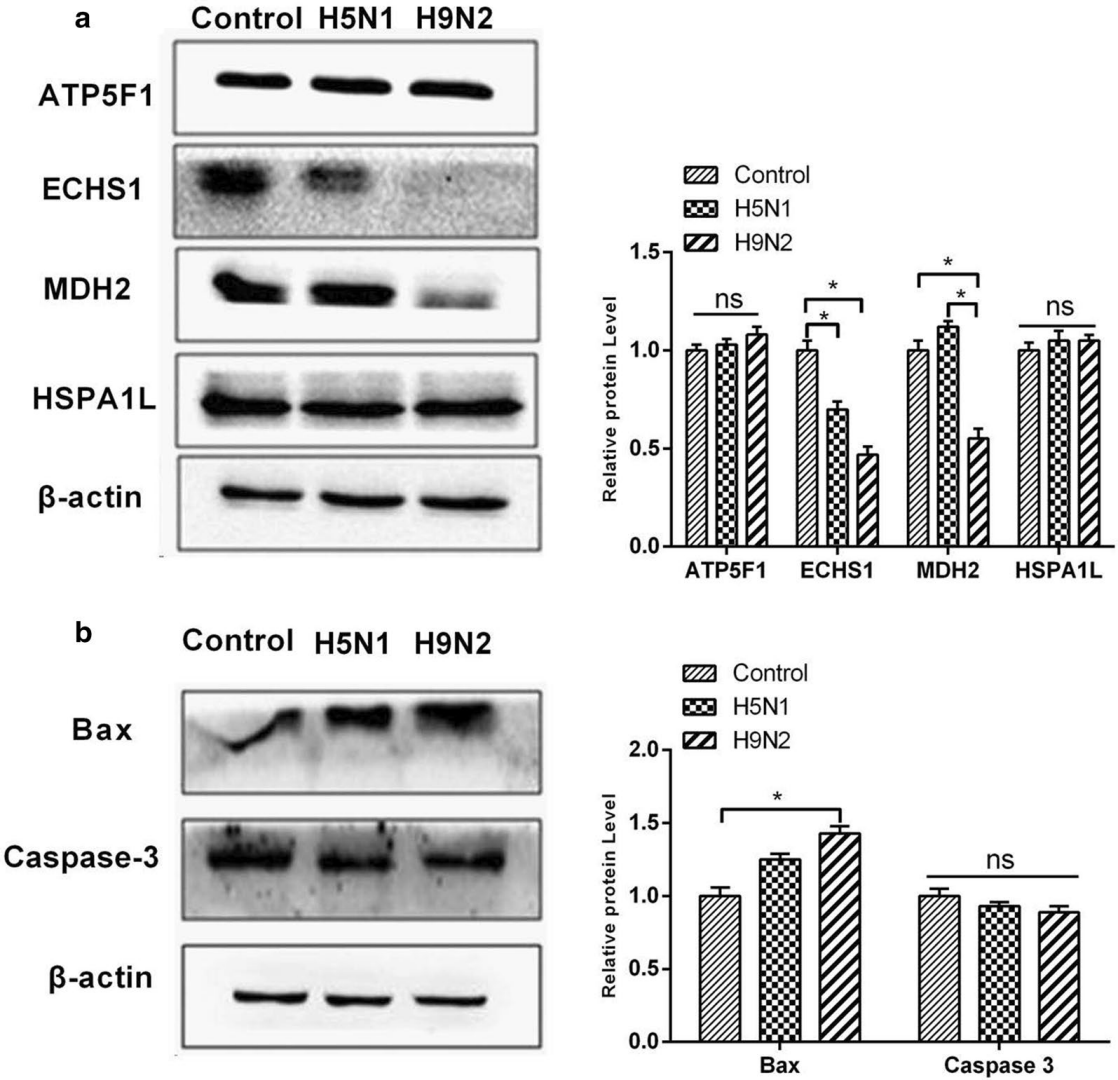
Western blot analysis of total protein. **a** Effects of H9N2 and H5N1 infection on ATP5F1, ECHS1, HSPA1L and MDH2 total protein expression in A549 cells. **b** Western blot analysis of BAX and Caspase 3 protein levels in normal A549 cells and H5N1- and H9N2-infected A549 cells. β-actin was used as an internal reference. *, *p* < 0.05; n = 3

To validate the results of the proteomic studies, we performed Western blot analysis of the differentially expressed mitochondrial proteins ATP5F1, ECHS1, MDH2, and HSPA1L with the TOM40 protein as the control. The results are shown in Fig. 4; the density of the band corresponding to the mitochondrial protein MDH2 was significantly reduced in the H5N1-infected group compared with that in H9N2-infected group (P < 0.05). In addition, the densities of the ECHS1 and HSPA1L protein bands were significantly enhanced (P < 0.05, for both). The ATP5F1 band was weak, but the difference was nonsignificant. We performed the same Western blot experiment with total cell protein, and the results are shown in Fig. 5a. There were significant differences between ECHS1 and MDH2 in the total protein.

ECHS1 is associated with apoptosis; we speculate that the difference between hosts infected with different subtypes of influenza virus may be related to apoptosis. Western blotting was used to assess the total protein levels of BAX and Caspase 3 in A549 cells, with β-actin as the internal reference. The results are shown in Fig. 5b. The expression of BAX was increased in both the H5N1-infected group and the H9N2-infected group and was slightly higher in the H9N2-infected group than in the H5N1-infected group. Caspase 3 (32 kDa) expression was reduced in both the H5N1-infected and H9N2-infected groups, but the difference between the two groups was nonsignificant. Total protein Western blotting experiments showed that the differentially expressed proteins were mainly related to endogenous apoptosis.

## Discussion

To date, many researchers have applied proteomics to study whole-cell proteomics during infection with influenza viruses such as H5N1, H3N2, and H1N1. However, we believe that compared with whole-cell proteomics, subcellular proteomics is more capable of identifying early diagnostic markers of influenza virus infection and is more conducive to the analysis of disease-related proteins and observation of the dynamic process of host cell infection with the virus. IAV can induce apoptosis [22], and the apoptotic pathway occurs in mitochondria [23]. To study the effect of infection with two different subtypes of influenza virus on the mitochondrial proteome, there is no need to set a blank control. We performed subcellular mitochondrial proteomic analysis of A549 cells infected with H5 and H9 subtype avian influenza viruses [24, 25]. To better study the process of viral infection of the host, we chose the 24 h time point to study the mitochondrial protein differences by two viruses with different pathogenicity [15].

Two-dimensional electrophoresis allows differential distribution of many protein isotypes. After data redundancy removal, we found that 16 proteins were upregulated and 16 were downregulated in the H5N1-infected group compared with those in the H9N2-infected group. However, further validation of the subcellular localization of some proteins is needed. Among the identified mitochondrial proteins, 6 mitochondrial proteins were downregulated and 2 mitochondrial proteins were upregulated in the H5N1-infected group compared with those in the H9N2-infected group.

Based on GO analysis, most differentially expressed proteins were binding proteins. A variety of binding proteins that affect the virulence of influenza viruses have been discovered, such as poly (rC)-binding protein 2 and nuclear export protein 1 [26, 27]. We found that these differentially expressed binding proteins may be related to the mechanism of influenza virus infection. Therefore, our findings are helpful for further analysis of the mechanism that binds proteins to influenza viruses.

Among the upregulated mitochondrial proteins was the molecular chaperone HSPA1L, a member of the 70-kDa heat shock protein (HSP70) family that is localized to the mitochondrial matrix and whose coding gene is located on chromosome 6p21 in the HLA class III region [28]. Other studies have shown that HSP70 appears to be upregulated during infection with HPAI virus compared to during infection with LPAI virus This chaperone is involved in a variety of cellular processes, including folding and transport of newly synthesized polypeptides, proteolytic activation of misfolded proteins, and formation and dissociation of protein complexes [29]. The ECHS1 protein is found in mitochondria, peroxisomes, and smooth endoplasmic reticulum. The upregulated protein enoyl-CoA hydratase, encoded by ECHS1 on chromosome 10, is a 160-kDa hexamer enzyme consisting of 290 amino acids and is located in the mitochondrial matrix. ECHS1 is associated with mitochondrial short-chain and medium-chain fatty acid β-oxidation and branched-chain amino acid catabolic pathways, as well as other catabolic pathways [30]. In the absence of hepatitis B virus infection, the ECHS1 gene was subjected to RNA interference and was found to promote apoptosis after transfection into HepG2 cells [31]. However, in Xiao et al.’s study in hepatitis B virus-infected HepG2 cells, ECHS1, a binding protein of hepatitis B virus surface antigen, promoted HepG2 cell apoptosis. The coexistence of ECHS1 and hepatitis B virus surface antigen changed the expression of Bcl-2 family proteins; specifically, 12 proapoptotic proteins were upregulated, and 8 antiapoptotic proteins were downregulated [32]. The results of this study are consistent with those obtained after RNA interference in the absence of hepatitis B virus infection, indicating that not all viruses can use ECHS1 as a binding protein for viral surface antigens, thereby promoting apoptosis. Related studies have confirmed that influenza virus can induce apoptosis. In our study, the difference in BAX expression detected by Western blotting showed that the level of endogenous apoptosis induced by HPAIV was higher than that induced by LPAIV. Endogenous apoptosis leads to mitochondrial swelling, disappearance of internal cristae and permeabilization, a possible reason for the difference in the virulence of these two viruses. In addition, downregulation of ECHS1 protein expression affects the fatty acid β oxidation pathway and reduces the replication ability of RNA viruses such as measles virus, vesicular stomatitis virus, and Semliki Forest virus [33]. Our results revealed that the expression of ECHS1 protein was upregulated in the H5N1 virus-infected group compared with that in H9N2 virus-infected group, which may explain why the H5N1 virus is more pathogenic than the H9N2 virus.

Among these downregulated mitochondrial proteins, the heat shock 70-kDa protein 1-like, malate dehydrogenase, mitochondrial membrane ATP synthase, and stomatin-like 2 proteins are located in the mitochondrial inner membrane, while the peroxiredoxin 5 and 60-kDa heat shock proteins are located in the mitochondrial matrix. HSPA1L indirectly affects body metabolism and biological function by regulating iron-sulfur protein maturation [34]; malate dehydrogenase is associated with the TCA cycle [4]; ATP synthase is involved in energy production and permeability transition pores (PTP, key players in cell death); stomatin-like protein 2 is involved in T cell activation, calcium homeostasis, and the stress response [35]; peroxiredoxin-5 plays an antioxidative stress role in cell protection [36, 37]; and 60-kDa heat shock protein is involved in controlling protein folding, the stress response, and the delivery of endogenous peptides to antigen presenting cells [38].

These eight differentially expressed mitochondrial proteins, especially ECHS1, may be used as new antiviral targets, but the results need to be further verified by a series of methods, such as RNA interference. In IAV proteomic studies by other groups, 60-kDa heat shock protein, 70-kDa heat shock protein and ATP synthase subunits often appear as differentially expressed proteins. Are these differentially expressed proteins commonly regulated by different IAVs? To date, a relatively small amount of proteomic data have been obtained for different IAVs. Thus, a large amount of proteomics data for different IAV infections is needed to analyze the common differentially expressed proteins for different IAV infections and the pathogenic mechanism of IAV.

We hypothesized that H5N1 is highly pathogenic compared with H9N2, probably because of the upregulation and downregulation of the above eight mitochondrial proteins, which in turn inhibit T cell activation, antigen presentation, stress responses, and other processes. The increased mortality from H5N1 infection may also be due to metabolic abnormalities. A total of 42.3% of these differentially expressed proteins were involved in the apoptotic process, and we speculate that the altered levels of mitochondrial protein expression during IAV pathogenesis are due mainly to the difference in the endogenous apoptotic process. Our analysis also identified many other influencing factors, indicating that host cell infection is a complex process.

## Conclusions

In this study, we infected A549 cells with H5N1 and H9N2 AIVs, then extracted the mitochondrial proteins of the infected cells for differential protein analysis. Through analysis of the functions of the differential proteins, it was found that compared with that in the H9N2 group, T cell activation in the H5N1 group was reduced and antigen presentation was weakened. Based on GO analysis, most of these differential proteins were related to apoptosis. To a certain extent, the pathogenicity of different IAVs is related to their ability to cause apoptosis. In our research, we identified different proteins, such as Stress-70 protein, peroxide reductase-5, enoyl-CoA hydratase, Stomatin-like protein 2, ATP synthase, and 60-kDa heat shock protein. These identified proteins play an important role in apoptosis. This study aids the analysis of the pathogenic mechanism of influenza viruses with different virulence, and provides a reference for the selection of anti-influenza virus host targets.

## Supplementary Information

**Additional file 1.** **Figure S1**: 2-DE gel images of H9N2-infected and H5N1 groups of A549 cells at 24 hpi. a 2-DE gel of the H5N1-infected group. b 2-DE gel of the H9N2-infected group. The distribution of differential protein spots identified by mass spectrometry in the two-dimensional electrophoresis pattern. The downward arrow indicates that H5N1 expressed down-regulated protein spots compared to the low-toxic control; the upward arrow indicates that H5N1 expressed up-regulated protein spots compared to the low-toxic control. c Enlarged regions of several differentially expressed protein spots. Differentially expressed protein spots are indicated by numbers and circles.

## Data Availability

The mass spectrometry proteomics data have been deposited to the ProteomeXchange Consortium (http://proteomecentral.proteomexchange.org) via the iProX partner repository, dataset identifier PXD017921.

## Abbreviations

AIV: Avian influenza virus
HPAI: Highly pathogenic avian influenza
LPAI: Low pathogenic avian influenza
2D: Two-dimensional difference gel electrophoresis
MALDI-TOF: Matrix-assisted laser desorption/ionization time of flight
GO: Gene ontology
KEGG: Kyoto encyclopedia of genes and genomes
FBS: Fetal bovine serum
BSA: Bovine serum albumin
EGTA: Ethylene glycol bis(2-aminoethyl)tetraacetic acid
HEPES: 4-(2-Hydroxyethyl)piperazine-1-ethanesulfonic acid
NCBI: National center for biotechnology information
MOI: Multiplicity of infection
PBS: Phosphate-buffered saline
SD: Standard deviation
