# Supplementary material for: Differential mitochondrial proteomic analysis of A549 cells infected with avian influenza virus subtypes H5 and H9
Source: Virol J. 2021 Feb 18;18:39. doi: 10.1186/s12985-021-01512-4 (PMC7891018; doi:10.1186/s12985-021-01512-4)
Supplement: Supplementary file 1 — Additional file 1. Figure S1: 2-DE gel images of H9N2-infected and H5N1 groups of A549 cells at 24 hpi. a 2-DE gel of the H5N1-infected group. b 2-DE gel of the H9N2-infected group. The distribution of differential protein spots identified by mass spectrometry in the two-dimensional electrophoresis pattern. The downward arrow indicates that H5N1 expressed down-regulated protein spots compared to the low-toxic control; the upward arrow indicates that H5N1 expressed up-regulated protein spots compared to the low-toxic control. c Enlarged regions of several differentially expressed protein spots. Differentially expressed protein spots are indicated by numbers and circles. [file 12985_2021_1512_MOESM1_ESM.doc]

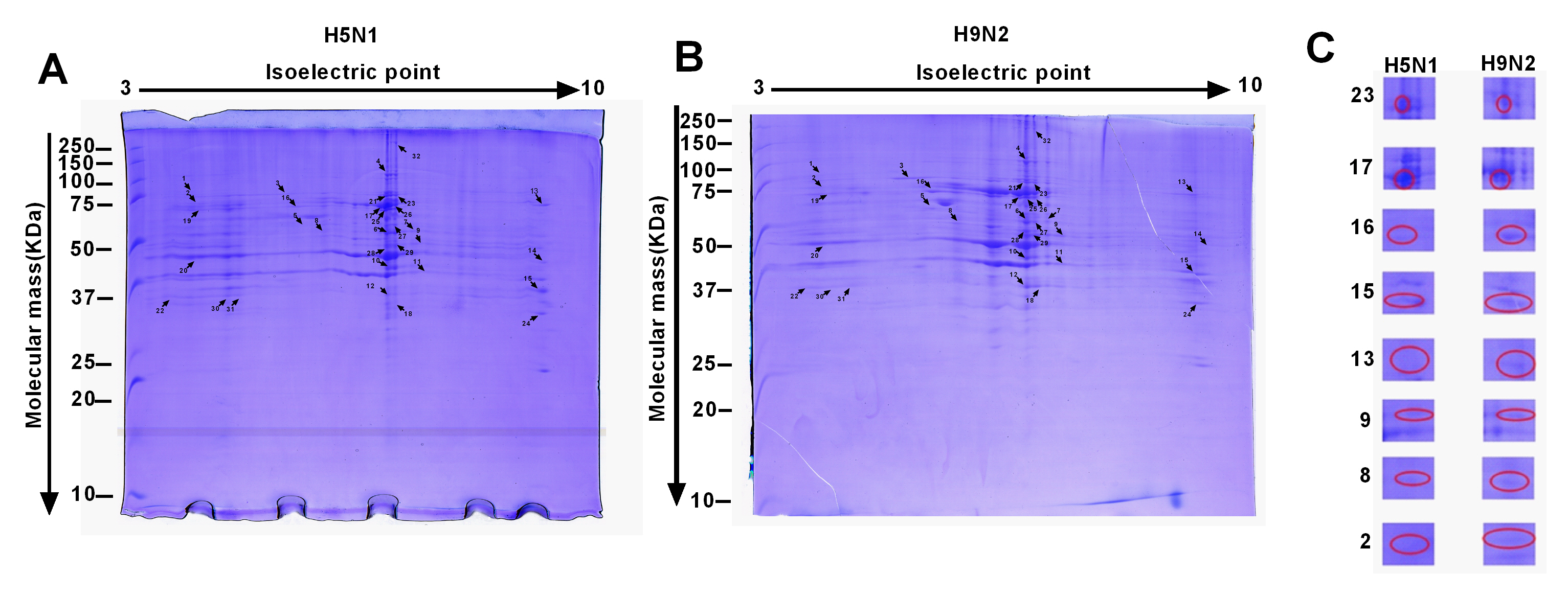


**Figure S1.** 2-DE gel images of H9N2-infected and H5N1 groups of A549 cells at 24 hpi.

**a** 2-DE gel of the H5N1-infected group. **b** 2-DE gel of the H9N2-infected group. The distribution of differential protein spots identified by mass spectrometry in the two-dimensional electrophoresis pattern. The downward arrow indicates that H5N1 expressed down-regulated protein spots compared to the low-toxic control; the upward arrow indicates that H5N1 expressed up-regulated protein spots compared to the low-toxic control. **c** Enlarged regions of several differentially expressed protein spots. Differentially expressed protein spots are indicated by numbers and circles.
